# Independent practice approaches for expressive piano performance: modeling, structural understanding, and narrative imagery

**DOI:** 10.3389/fpsyg.2026.1836640

**Published:** 2026-06-08

**Authors:** Su-Young Bae

**Affiliations:** Graduate School of Education, Sungshin Women’s University, Seoul, Republic of Korea

**Keywords:** expressive performance, music pedagogy, music practice, modeling, structural understanding, narrative imagery

## Abstract

This study compared three practice approaches for expressive performance—modeling, structural understanding, and narrative imagery—among 54 undergraduate and graduate piano majors. Participants were randomly assigned to one of the three practice conditions and completed a pretest–practice–posttest protocol using the opening excerpt from Chopin’s *Nocturne in E-flat major*, Op. 9, No. 2. During a 60-min practice session, participants practiced according to their assigned condition while annotating their scores and recording and reviewing their performances. Pretest and posttest performances were evaluated by three expert pianists across multiple dimensions, including phrasing, tone color, dynamics, tempo rubato, balance, articulation, overall expressiveness, and accuracy. Participants also completed a postsession questionnaire assessing goal clarity, perceived improvement, cue awareness, personal interpretation, engagement, and transfer intention. Mixed-design ANOVAs revealed significant Time × Group interactions for tone color, dynamics, tempo rubato, and articulation. The modeling group showed greater improvement than the narrative imagery group in tone color, tempo rubato, and articulation, and both the modeling and structural understanding groups showed greater improvement than the narrative imagery group in dynamics. Learner-reported outcomes, however, favored structural understanding and narrative imagery. Specifically, narrative imagery yielded higher perceived improvement than modeling, and both structural understanding and narrative imagery yielded higher cue awareness, personal interpretation, and transfer intention than modeling. Goal clarity and engagement did not differ significantly across groups. Open-ended responses indicated that modeling heightened awareness of specific expressive cues but was sometimes experienced as restrictive, whereas narrative imagery was reported to enhance engagement and interpretive agency. Taken together, these findings suggest that expressive performance is multidimensional and that different practice approaches may support different aspects of expressive development, with implications for how expressive performance is taught and evaluated comprehensively.

## 1 Introduction

In Western classical music, expressive performance extends beyond the accurate realization of notated information. Although notation provides a framework for pitch, rhythm, and certain expressive indications, such as dynamics and articulation, it does not fully determine how expressive details are to be realized in performance (Clarke, 1988; Gabrielsson, 1999; Gibbs, 2015; Howat, 1995; Palmer, 1997). Performers must therefore shape expressive timing, rubato, tone color, phrasing, and dynamic contour in relation to the musical context, thereby making structure, character, and interpretive intention audible to listeners (Clarke, 1988; Doğantan-Dack, 2014; Héroux, 2016, 2018; Juslin and Persson, 2002; Meissner, 2021; Palmer, 1997; Payne, 2016). From this perspective, expressive performance depends not only on technical control but also on the performer’s capacity to communicate musical meaning through sound.

To make such expressive decisions, performers need a clear internal representation of the intended performance. Drawing on research on expert music performance, Lehmann and Ericsson (1997) proposed a model of mental representations underlying skilled musical action. Adapting this framework to expressive performance, Woody (2003) articulated three component cognitive skills: goal imaging, motor production, and self-monitoring. Of these, goal imaging is especially important because it provides the internal reference against which performers compare their actual playing and make adjustments. When performers can relate their current performance to a clear goal image, they are better able to detect discrepancies and modify motor production accordingly (Woody, 2003). This emphasis aligns with later work on music practice and self-regulated learning. From a self-regulated learning perspective, goal setting is a subprocess of task analysis in the forethought phase and supports subsequent monitoring and strategic adjustment during practice (McPherson and Zimmerman, 2011). Likewise, McPhail (2025) emphasizes that learners need both a clear sense of the intended outcome and an awareness of the gap between that ideal and their current performance so that practice can be guided more purposefully.

Research on expressive performance pedagogy has proposed several approaches to help learners form and refine such internal representations, including aural modeling, inquiry and discussion about musical character, and imagery- or metaphor-based instruction (Meissner, 2021; Meissner and Timmers, 2019). The present study focuses on three approaches that can be self-applied during independent practice: modeling, structural understanding, and narrative imagery. These approaches are of particular interest because they represent different routes to specifying the intended performance: externally through an aural model, analytically through musical structure, and imaginatively through narrative imagery.

Modeling offers an external aural reference for the intended performance. This approach typically involves listening to live or recorded expert performances in order to build internal aural representations of the music and guide expressive decisions (Hallam, 1997, 1998; Meissner, 2021; Sloboda, 2005). Such models can provide learners with an aural picture of the music and support phrasing, expressiveness, accuracy, and technical fluency (Frewen, 2010; Meissner, 2021; Meissner and Timmers, 2020; Rosenthal, 1984), particularly when modeling is combined with inquiry and discussion about interpretation (Hallam, 1998; Meissner, 2021; Meissner and Timmers, 2020). Woody’s (2003) findings further suggest that modeling may be more effective when learners actively process what they hear: pianists who could verbally describe expressive features in an aural model reproduced them more accurately, especially for less idiomatic features. More recent work has likewise emphasized that recordings help learners establish a performance goal, perceive the gap between current and intended performance, and guide error detection and self-correction during practice (McPhail, 2025). At the same time, modeling raises pedagogical questions about possible dependence on external models and the extent to which learners are encouraged to develop their own interpretations (Meissner, 2021; Volioti and Williamon, 2017).

Structural understanding offers a different route to specifying the intended performance. Rather than beginning from an external aural model, this approach grounds expressive decisions in musical structure, including phrase organization, harmonic motion, tension and release, and points of climax. Research on expressive performance has long suggested that musical structure is not merely something to be analyzed but something to be communicated in sound: performers use timing, dynamics, articulation, and related cues to project phrase boundaries, grouping, accent patterns, and harmonic direction (Clarke, 1988; Friberg and Battel, 2002; Palmer, 1996, 1997; Repp, 1992, 1998). Structural understanding can therefore serve as a practical basis for shaping phrasing and expressive contour rather than as theoretical knowledge alone (Friberg and Battel, 2002), and performers’ interpretations of structure directly influence the expressive qualities of performance (Friberg and Battel, 2002; Palmer, 1989, 1996).

Narrative imagery offers a third route to specifying the intended performance. This approach draws on extra-musical associations such as moods, characters, motion, and imagined scenes to clarify expressive intention. Research on expressive performance has long shown that musical expression may be understood and communicated in relation to emotion, character, and movement (Gabrielsson and Juslin, 1996; Juslin, 2003; Juslin and Persson, 2002; Juslin and Timmers, 2010; Meissner, 2021). In performance pedagogy, metaphor, imagery, and story-like prompts are often used to suggest what the music should sound like (Brenner and Strand, 2013; Lindström et al., 2003), and such devices may help learners translate abstract expressive intentions into concrete musical actions (Schippers, 2006; Woody, 2002, 2006a,2006b). Related work on performers’ creative processes likewise suggests that extra-musical supports, including images, memories, analogies, and narratives, can inform interpretive decision-making and shape performers’ experience of nuance, phrasing, sonority, and musical character (Héroux, 2016; Héroux et al., 2020). In the present study, narrative imagery is treated as one specific form within this broader imagery- and analogy-based route.

At the same time, the effects of expressive strategies cannot be adequately understood from a single evaluative perspective. Given the multidimensional nature of expressive performance (Fabian et al., 2014), performance profiles may vary depending on the focus of the instruction or intention that guides the performer. Van Zijl et al. (2014), for example, showed that technical, expressive, and emotional performance instructions yielded distinct performance characteristics: expressive performances were more projected and audience-directed (faster tempo, louder sound, and wider dynamic range), whereas emotionally focused performances were more introverted and personal (slower tempo, softer sound, and narrower vibrato). A discrepancy may also arise between the performer’s subjective internal experience and the musical result as perceived by listeners. In Van Zijl et al. (2014), six of the eight performers regarded the emotional condition as their best performance; however, they also noted that intense internal emotions could distort their perception of the auditory quality. Listener research revealed a different evaluative pattern: in Van Zijl and Luck (2013), audiences preferred the expressive performances and judged them as more skilled, whereas emotional performances were rated as most expressive of sadness. Héroux et al. (2020) likewise reported in a pilot study that a condition based on notated musical indications was judged more expressive and more appreciated than an extra-musical condition, while also noting that performers’ technical weaknesses made expert judgments of expressiveness difficult. Taken together, these findings suggest that the effects of different approaches to expressive performance may not be adequately captured by a single global measure. Accordingly, the effectiveness of such strategies should be examined through an integrated lens that includes both multidimensional external evaluations across musical elements and learners’ subjective internal experiences.

A related gap in the literature concerns the context in which expressive strategies have been examined. Existing research on expressive performance has focused largely on teaching contexts rather than on learners’ independent practice (Meissner, 2021), even though students often rely on whole-piece play-throughs and naive repetition instead of more targeted, self-regulated practice strategies (Hallam, 1997, 2001a,2001b; McPhail, 2025; Mornell et al., 2020; Pitts and Davidson, 2000). Reviews and theoretical discussions have therefore emphasized the importance of more intentional and self-regulated approaches to practice (McPhail, 2025; McPherson and Zimmerman, 2011; Miksza, 2011). Yet, relatively little is known about the comparative efficacy of expressive strategies when learners apply them in independent practice.

To address this research gap, the present study compared three approaches to expressive performance in independent practice among university-level piano majors: modeling, structural understanding, and narrative imagery. Using an integrated evaluative approach, the study examined whether these approaches differed in expert-rated change across multiple musical dimensions and in learners’ reported practice experiences. The research questions were as follows:

(1) How do modeling, structural understanding, and narrative imagery differ in their effects on multidimensional changes in expressive performance during independent practice?
(2) How do these approaches differ in learners’ reported experiences of independent practice, including perceived improvement and perceived usefulness for future practice?

## 2 Materials and methods

### 2.1 Participants

Fifty-four undergraduate and graduate piano performance majors at a women’s university in Seoul, South Korea, participated voluntarily. All participants were women. The sample comprised 45 undergraduates and 9 graduate students, ranging in age from 19 to 29 years (*M* = 22.76, *SD* = 2.61). Participants were recruited through university bulletin boards and departmental group messaging platforms. They were stratified by academic level (undergraduate vs. graduate) and then randomly assigned to one of three strategy-guided practice conditions: the modeling condition, the structural understanding condition, and the narrative imagery condition, yielding three groups of 18 participants each. For brevity, these conditions are hereafter referred to as modeling, structural, and imagery. All participants provided informed consent, and the study protocol, recruitment, consent, and data-handling procedures were approved by the university’s institutional review board.

### 2.2 Apparatus and materials

All sessions were conducted in a large practice room using identical Yamaha P-515 digital pianos. Because multiple participants used the room simultaneously, headphones were used throughout the session to minimize mutual acoustic interference and allow each participant to work and play independently. Performances were recorded as MIDI on the Yamaha P-515, enabling standardized playback and subsequent audio-file generation for expert ratings.

The task material was the opening excerpt of Chopin’s *Nocturne in E-flat major*, Op. 9, No. 2 (upbeat and mm. 1–8). The excerpt was selected because it is brief, musically coherent, and contains salient expressive markings in the score, providing a common basis for interpretation across conditions. To prepare listening stimuli for expert ratings, each participant’s MIDI recording was converted to an audio file during playback on the same Yamaha P-515 using the instrument’s MIDI-to-Audio function. Instrument settings relevant to playback and sound output were held constant across all conversions. The converted audio files were saved as WAV files (44.1 kHz, 16-bit, stereo). All files were reviewed to confirm the absence of truncation, clipping, or audible distortion. Filenames were anonymized and presentation order was randomized. Raters were blinded to the assigned condition and time point.

### 2.3 Research design and procedure

The study used a three-group pretest–posttest design with parallel strategy-guided practice conditions. Sessions were conducted under standardized conditions (i.e., same room and identical equipment). Due to room capacity, participants completed the protocol in subgroups of up to six at a time while practicing individually.

#### 2.3.1 Pretest

At the start of the session, participants received the score for the target excerpt and practiced individually for approximately 20–30 min. No condition-specific guidance was provided during this phase. Participants then recorded multiple takes on their assigned Yamaha P-515 and selected the take they considered their best. This best-effort take served as the pretest performance.

#### 2.3.2 Practice phase (strategy-guided practice)

Immediately after the pretest, participants completed 60 min of individual practice under their assigned condition. Because the excerpt was brief, practice time could be devoted primarily to the formation, testing, and refinement of expressive goals rather than to extensive note learning. Practice duration was held constant across conditions to control time on task. At the outset, the researcher provided brief condition-specific instructions and ensured adherence to the assigned materials and procedures; no further musical coaching, feedback, or corrective cues were provided during practice. Participants were allowed brief self-paced breaks within the 60-min practice period to minimize fatigue. In all conditions, participants were encouraged to annotate the score in ways that supported implementation of the assigned approach. They were also permitted to make interim recordings of their own playing, listen back as needed, and revise their performance accordingly. This recording-and-playback procedure was available as a self-monitoring resource in all conditions.

In the modeling condition, participants used a professional audio recording of the excerpt provided on a tablet and listened to it over headphones as needed. They were instructed to use the model as an external auditory reference and to align their own performance with salient expressive features of the model, such as phrasing, timing and rubato, dynamic contour, and tone color. Participants were encouraged to annotate salient cues on the score for use during practice.

In the structural condition, participants received a brief handout summarizing structural features of the excerpt, including phrase segmentation as indicated by the score, salient melodic motion, harmonic progression, and tension–release and climactic points, together with indications of how notated dynamics might be interpreted in relation to these features. They were instructed to derive expressive decisions from these structural relations and to record relevant structural cues and planned expressive actions on the score. Participants were encouraged to use segment-based practice, harmonic clarification (e.g., chord blocking), and coordination of touch and pedaling with identified structural goals to support this process.

In the imagery condition, participants received a brief narrative prompt aligned with the excerpt’s overall character. For each phrase segment, participants generated a brief image, scene, or one-sentence scenario and wrote it on the score. They then practiced shaping timing, dynamics, tone color, and phrasing so that the imagined progression was conveyed across the passage.

#### 2.3.3 Posttest

Immediately after the practice phase, participants repeated the same recording procedure on their assigned Yamaha P-515 and selected the take they considered their best, just as they had done during the pretest. This take served as the posttest performance.

### 2.4 Measures

#### 2.4.1 Expert ratings of performance

Pretest and posttest performances were evaluated by three expert pianists (DMA-level), active as university faculty and professional performers. Raters received only anonymized audio files identified by random codes and were blinded to condition and time point. The file order was randomized such that the two performances from the same participant did not appear consecutively. Prior to scoring, raters reviewed the rating rubric to align the interpretation of scale anchors.

Each recording was rated on a 5-point scale (1 = very poor, 5 = excellent) for nine dimensions: phrasing, tone color, dynamics, tempo rubato, balance, pedaling, articulation, overall expressiveness, and accuracy. Interrater reliability was assessed using average-measures intraclass correlation coefficients (ICCs) based on a two-way mixed-effects model with consistency. ICCs were 0.801 for phrasing, 0.752 for tone color, 0.804 for dynamics, 0.794 for tempo rubato, 0.753 for balance, 0.335 for pedaling, 0.770 for articulation, 0.776 for overall expressiveness, and 0.782 for accuracy. Because pedaling showed low interrater reliability and pedal-related cues were difficult to judge consistently from standardized digital-piano audio, pedaling was excluded from inferential analyses and reported descriptively. For inferential analyses, ratings were averaged across the three raters for each remaining dimension.

#### 2.4.2 Student questionnaire

Immediately after the posttest, participants completed a short questionnaire assessing their experience of the assigned practice strategy using a 5-point Likert scale (1 = strongly disagree, 5 = strongly agree). Items assessed included: (a) goal clarity/goal imaging (“I had a clear idea of how I wanted the music to sound during practice”), (b) perceived improvement in expressiveness (“I felt that my expressive performance improved through this practice”), (c) cue awareness (“I became more aware of structural and expressive features in the music, e.g., phrase shape, tension–release points, dynamics, timing”), (d) personal interpretation and ownership (“The strategy helped me create a personal interpretation of the piece”), (e) engagement and focus during practice (“I was able to stay engaged and focused during practice”), and (f) transfer intention (“I would consider applying this strategy in future practice”). Because each construct was assessed with a single item, questionnaire responses were analyzed at the item level rather than combined into a total scale. An open-ended question asked participants to describe any notable thoughts or feelings experienced during practice.

### 2.5 Data analysis

For expert ratings, descriptive statistics (means and standard deviations) were computed for each group at pretest and posttest. For each performance dimension, a mixed-design ANOVA was conducted with Time (Pre, Post) as the within-subject factor and Group (modeling, structural, imagery) as the between-subject factor. Partial eta squared (η_p_^2^) was reported as an effect size. For dimensions showing a significant Time × Group interaction, follow-up one-way ANOVAs on change scores (Δ = Post–Pre) were conducted, followed by Tukey HSD post hoc tests.

For questionnaire items, one-way ANOVAs were conducted to test group differences, followed by Tukey HSD post hoc tests where applicable. Homogeneity of variance across groups was assessed using Levene’s test before interpreting the one-way ANOVA and Tukey HSD results. Open-ended responses were summarized using a brief qualitative content analysis approach to identify recurring categories within each condition and to support interpretation of the quantitative findings. All analyses were conducted using IBM SPSS Statistics 28.

## 3 Results

### 3.1 Expert-rated performance outcomes

#### 3.1.1 Descriptive statistics

Table 1 summarizes mean change scores (Δ = Post–Pre) for each expert-rated dimension by group (*n* = 18 per group; see Supplementary Table 1 for detailed descriptive statistics). Positive values indicate improvement from pretest to posttest. Descriptively, modeling (G1) and structural (G2) showed positive mean change on several dimensions, whereas imagery (G3) showed little or negative mean change in several dimensions, including tone color, dynamics, tempo rubato, and articulation. Because interrater reliability for pedaling was low (average-measures ICC = 0.335), pedaling is reported descriptively only and was excluded from inferential analyses.

**TABLE 1:** Mean change scores (Δ = Post–Pre) for expert-rated performance dimensions by group.

Measure	Modeling (G1) *M* (*SD*)	Structural (G2) *M* (*SD*)	Imagery (G3) *M* (*SD*)
Phrasing	0.26 (0.62)	0.39 (0.49)	–0.06 (0.54)
Tone color	0.33 (0.57)	0.11 (0.36)	–0.28 (0.51)
Dynamics	0.28 (0.46)	0.22 (0.47)	–0.28 (0.51)
Tempo rubato	0.46 (0.49)	0.41 (0.53)	–0.02 (0.64)
Balance	0.04 (0.39)	0.15 (0.54)	0.15 (0.53)
Pedaling†	–0.07 (0.58)	0.11 (0.26)	0.06 (0.49)
Articulation	0.33 (0.54)	0.19 (0.26)	–0.07 (0.37)
Overall expressiveness	0.04 (0.81)	0.39 (0.38)	–0.04 (0.74)
Accuracy	0.13 (0.53)	–0.24 (0.36)	–0.11 (0.62)

*n* = 18 per group. †Pedaling is reported descriptively only (ICC = 0.335) and excluded from inferential analyses.

#### 3.1.2 Mixed-design ANOVAs

Mixed-design ANOVAs (Time: Pre/Post × Group: G1–G3) were conducted for each expert-rated dimension, excluding pedaling (Table 2; see also Supplementary Table 2). A significant main effect of Time was found for phrasing, indicating overall improvement from pretest to posttest across groups. Significant main effects of Time were also found for tempo rubato and articulation; however, these should be interpreted in light of the significant Time × Group interactions. Significant Time × Group interactions were observed for tone color, dynamics, tempo rubato, and articulation.

**TABLE 2:** Mixed-design ANOVA results for expert-rated performance dimensions.

Measure	Time			Time × Group		
	*F*(1, 51)	*p*	η_p_^2^	*F*(2, 51)	*p*	η_p_^2^
Phrasing	6.903	0.011	0.119	3.080	0.055	0.108
Tone color	0.692	0.409	0.013	7.154	0.002	0.219
Dynamics	1.271	0.265	0.024	7.229	0.002	0.221
Tempo rubato	14.041	<0.001	0.216	4.028	0.024	0.136
Balance	2.765	0.102	0.051	0.307	0.737	0.012
Articulation	7.200	0.010	0.124	4.650	0.014	0.154
Overall expressiveness	2.021	0.161	0.038	2.076	0.136	0.075
Accuracy	1.135	0.292	0.022	2.434	0.098	0.087

η_*p*_^2^ = partial eta squared. Pedaling was excluded from inferential analyses because interrater reliability was low.

#### 3.1.3 Post hoc tests

To interpret significant interactions, Tukey HSD tests were conducted on change scores (Table 3; see also Supplementary Table 3). The results showed that modeling improved more than imagery in tone color (*p* = 0.001), tempo rubato (*p* = 0.033), and articulation (*p* = 0.011). For dynamics, both modeling and structural improved more than imagery (*p*s = 0.003 and 0.009, respectively). No significant differences were observed between modeling and structural for any outcome with a significant interaction, and all remaining pairwise comparisons were nonsignificant.

**TABLE 3:** Tukey HSD post hoc comparisons of change scores for significant Time × Group interactions.

Measure	Pairwise comparison	Mean difference	*p*
Tone color	G1-G3	0.611	0.001
Dynamics	G1-G3	0.556	0.003
Dynamics	G2-G3	0.500	0.009
Tempo rubato	G1-G3	0.481	0.033
Articulation	G1-G3	0.407	0.011

Comparisons are based on change scores (Δ = Post–Pre). G1 = modeling, G2 = structural, G3 = imagery.

### 3.2 Student-reported questionnaire outcomes

#### 3.2.1 Closed-ended items

Table 4 presents descriptive statistics and one-way ANOVA results for postsession questionnaire items. Group differences were significant for perceived improvement, cue awareness, personal interpretation, and transfer intention (*p*s ≤ 0.003), whereas goal clarity and engagement did not differ significantly across groups (*p*s ≥ 0.115). Tukey HSD tests showed that perceived improvement was higher in imagery than in modeling (*p* = 0.002). For cue awareness, personal interpretation, and transfer intention, both structural and imagery scored higher than modeling (all *p*s ≤ 0.011), with no significant differences between structural and imagery.

**TABLE 4:** Student questionnaire responses by group and one-way ANOVA results.

Construct	Modeling	Structural	Imagery	*F*(2, 51)	*p*	Significant Tukey HSD comparisons
	*M* (*SD*)	*M* (*SD*)	*M* (*SD*)			
Goal clarity	4.39 (0.61)	4.50 (0.51)	4.06 (0.80)	2.261	0.115	—
Perceived improvement	3.78 (0.65)	4.22 (0.65)	4.50 (0.51)	6.507	0.003	G1 < G3
Cue awareness	3.89 (0.58)	4.44 (0.51)	4.44 (0.51)	6.439	0.003	G1 < G2, G1 < G3
Personal interpretation	3.44 (0.62)	4.39 (0.61)	4.33 (0.49)	15.419	<0.001	G1 < G2, G1 < G3
Engagement	4.50 (0.51)	4.22 (0.73)	4.44 (0.78)	0.824	0.444	—
Transfer intention	4.00 (0.69)	4.78 (0.43)	4.56 (0.51)	9.471	<0.001	G1 < G2, G1 < G3

G1 = modeling, G2 = structural, G3 = imagery.

#### 3.2.2 Open-ended responses

Open-ended responses were summarized using a brief qualitative content analysis approach and are detailed in Table 5. In the modeling condition, participants frequently reported heightened awareness of fine-grained expressive cues and described using the model as a reference point for evaluating and shaping their own performance. At the same time, some participants experienced the process as cognitively demanding, time-intensive, or constraining to their sense of personal expression. In the structural condition, participants highlighted improved understanding of harmonic change, phrase flow, and structural relations, which supported more deliberate expressive decisions, although some reported reduced naturalness and physical discomfort when analytical focus became excessive. In the imagery condition, participants described stronger emotional engagement, richer expressive ideas, and clearer interpretive direction through storytelling, while also noting initial difficulty in generating a narrative and translating it into sound.

**TABLE 5:** Summary of categories identified in open-ended responses by practice condition.

Condition	Category	Brief description	Illustrative quote(s)
Modeling	Expressive cue awareness	Participants reported heightened awareness of detailed expressive cues, including phrasing, rubato, accents, and balance, through close listening to and imitation of the model performance.	“I noticed accents, breathing, and phrasing details I had previously missed.” (M04)
	Self-evaluation and goal setting	Participants used the model as a reference point for evaluating their own playing, clarifying expressive goals, and generating ideas for shaping performance.	“Modeling helped me understand the musical flow better, and it was valuable to compare the performer’s interpretation with my own.” (M16)
	Cognitive load and time demands	Participants described the modeling process as cognitively demanding because multiple expressive elements had to be processed simultaneously, and also as difficult to internalize within a limited practice period.	“At first it felt complicated because there were too many specific things to imitate.” (M09) “Practicing through modeling within the time available felt somewhat difficult.” (M15)
	Reduced expressive immersion and ownership	Some participants felt that close imitation reduced immersion in their own expression and constrained their sense of personal phrasing or interpretive ownership.	“Although following the performer exactly would probably improve my playing, I felt less immersed in conveying my own emotions.” (M12) “In terms of phrasing, I found myself tending to abandon my own phrasing and follow the model’s phrasing unconditionally.” (M11)
Structural	Structural understanding and decision-making	Participants reported that awareness of harmonic change, phrase flow, and structural relations helped them understand the passage more clearly and make more deliberate expressive decisions.	“Feeling the harmonic changes helped me understand the flow of the piece.” (S02) “Through this analytical approach, I realized that there were harmonic changes and meanings even in passages I had previously passed over without much thought, and I was able to practice those parts with greater attention.” (S18)
	Reduced naturalness under analytic focus	Some participants felt that when analysis became too foregrounded, the playing felt less natural and was accompanied by physical discomfort.	“Perhaps because I was too focused on expressing the analysis, the playing felt less natural, and I also felt some physical discomfort while playing.” (S11)
Imagery	Emotional engagement and expressive ideas	Participants described stronger emotional engagement, more natural emergence of expressive detail, and richer expressive ideas through phrase-specific storytelling.	“When I gave each phrase a concrete story, detailed expressive elements seemed to emerge more naturally.” (I07)
	Interpretive direction and agency	Participants reported that storytelling helped clarify the intended expressive direction of the performance and increased their sense of agency in shaping interpretation.	“It was helpful because I could determine the direction of the performance I wanted to create.” (I14)
	Difficulty with narrative generation	Some participants reported initial difficulty creating a narrative image and translating it into sound.	“It was difficult to think of a story and to realize that story in sound, but in the process of finding a solution, ideas about expression began to emerge.” (I01)

## 4 Discussion

### 4.1 Overview of the main findings

The present study compared three self-applied approaches to expressive performance in independent piano practice—modeling, structural understanding, and narrative imagery—and examined how they differed in expert-rated performance change and in students’ reported practice experiences. The overall pattern of findings suggests that the three approaches did not support a single, uniform dimension of “better expressiveness.” Rather, they seemed to operate through different pathways and produce different kinds of outcomes. In the expert ratings, modeling showed greater improvement than imagery in tone color, tempo rubato, and articulation, while both modeling and structural showed greater improvement than imagery in dynamics. By contrast, in the questionnaire, imagery yielded higher perceived improvement than modeling, and both structural and imagery scored higher than modeling on cue awareness, personal interpretation, and transfer intention. At the same time, goal clarity and engagement did not differ significantly across conditions, suggesting that all three approaches helped students establish a relatively clear practice aim, while differing in how that aim was elaborated and translated into performance.

Taken together, these findings suggest that the three approaches may have supported expressive performance at different levels of regulation rather than along a single continuum of effectiveness. In this respect, the present results are compatible with prior work indicating that different performance foci may give rise to distinct performance profiles. Van Zijl et al. (2014), for example, showed that different instructional foci produced different auditory characteristics rather than a single uniformly optimal outcome, and related listener research (Van Zijl and Luck, 2013) further suggested that performers’ subjective experience does not necessarily coincide with what listeners judge to be more skilled or more effective. Similarly, the present findings suggest that modeling may have operated most directly at the level of externally guided cue adjustment, structural at the level of musically grounded organization of performance decisions, and imagery at the level of expressive intention and interpretive ownership. The relatively weaker immediate expert-rated outcomes of imagery are also compatible with Héroux et al. (2020), who reported that an extra-musical condition was less positively judged than a condition based on notated musical indications. From this perspective, the three approaches did not simply differ in overall effectiveness; rather, they appeared to support different kinds of expressive work within short independent practice.

### 4.2 Modeling: immediate cue-level gains but lower interpretive ownership

One of the clearest findings of the present study was that modeling was comparatively effective for short-term cue-level change, particularly in tone color, tempo rubato, articulation, and dynamics. This pattern is consistent with literature suggesting that aural modeling can provide learners with a relatively clear auditory target and help them perceive the gap between current and intended performance, thereby supporting rapid adjustment and self-correction during practice (Frewen, 2010; McPhail, 2025). In this sense, modeling may have been effective in the present study because it offered an immediately available external reference that could be translated into audible change within a short practice period. This interpretation is also compatible with previous work showing that imitative listening can exert a strong short-term influence on performance, even if only selected aspects of that influence are retained over time (Lisboa et al., 2005).

At the same time, the value of modeling did not appear to lie simply in trying to imitate an entire performance more closely. In the questionnaire, modeling was rated lower than both structural and imagery on cue awareness, personal interpretation, and transfer intention, and the open-ended responses help explain why. Students did report becoming more aware of details such as accents, breathing, phrasing, rubato, timing, and balance, and several responses suggested that the model helped them notice weaknesses or omissions in their own playing. However, they also described the process as complex and difficult when too many expressive elements were encountered at once. This pattern is noteworthy because, although modeling produced immediate gains on some expert-rated dimensions, students’ comments suggest that attempting to absorb an entire model holistically could lead to cognitive overload. From this perspective, the present findings suggest that modeling was most useful not when students tried to reproduce everything at once, but when it helped them identify and refine specific expressive details.

Beyond these cognitive challenges, another concern relates to interpretive ownership. Some students reported that close imitation drew them away from their own phrasing or reduced immersion in their own expression, which helps explain why modeling was less preferred for personal interpretation and future use despite its immediate cue-level benefits. This pattern is compatible with broader literature noting that imitation through recordings remains contested within Western art-music practice because of concerns about dependence, originality, and performance individuality (Volioti and Williamon, 2017). At the same time, the empirical findings of Volioti and Williamon (2017) suggest that listening need not suppress individuality if it is treated as a resource for comparison, evaluation, and decision making rather than as a demand for exact replication. The pedagogical implication, therefore, is not that modeling should be avoided, but that it should be used more selectively and reflectively. In short independent practice, modeling may be most productive when students focus on one or two musical elements at a time, such as rubato, articulation, or dynamic shaping, and when recordings are treated as interpretive examples rather than templates to be copied wholesale. More broadly, comparing multiple recordings may help expand interpretive options and support a more agentic use of modeling, allowing students to learn from external examples without relinquishing ownership of their own performance decisions.

### 4.3 Structural understanding: musically grounded organization of expression

Structural understanding also produced meaningful results. In the expert ratings, it did not differ significantly from modeling, and in dynamics, it showed greater improvement than imagery. This pattern suggests that expressive performance can be organized not only through an external auditory example but also through attention to the music’s internal organization, including phrase structure, harmonic motion, tension and release, and points of climax. This interpretation is consistent with long-standing work arguing that musical structure is not merely something to be analyzed, but something to be communicated in sound: performers use timing, dynamics, articulation, and phrasing to project grouping, accent patterns, phrase boundaries, and harmonic direction to the listener (Clarke, 1988; Friberg and Battel, 2002; Palmer, 1989, 1996, 1997; Repp, 1992, 1998). In this sense, the present findings support the view that performers’ interpretations of structure can directly shape expressive quality rather than simply provide conceptual knowledge about the score (Friberg and Battel, 2002; Palmer, 1989, 1996).

At the same time, some students reported that when analytical content became too foregrounded, the playing felt less natural and physically less free. This suggests that structural work is most useful not when analysis becomes an end in itself, but when it remains closely tied to sounding performance. From a pedagogical perspective, the implication is not simply that students need more analysis, but that harmonic and structural understanding should be integrated into practice as a basis for phrasing, dynamic shaping, and musical direction. Used in this way, structural understanding may help students move beyond surface note production toward more intentional and musically justified expressive decisions, while avoiding the loss of naturalness that can arise when analysis is treated as something separate from performance.

### 4.4 Narrative imagery: stronger subjective gains than immediate expert-detected change

Narrative imagery showed the clearest divergence between expert-rated change and students’ experienced learning. In the expert ratings, imagery did not show an advantage over the other two conditions and was relatively weaker on some cue-level dimensions, including tone color, dynamics, tempo rubato, and articulation. However, in the questionnaire, imagery received higher ratings than modeling on perceived improvement, cue awareness, personal interpretation, and transfer intention, and the open-ended responses likewise suggested stronger emotional engagement, richer expressive ideas, and clearer interpretive direction. Taken together, these findings suggest that imagery was particularly effective for strengthening students’ internal sense of expressive intention and ownership, even when such gains were not immediately converted into cue-level changes that expert listeners could detect after a short practice session. This interpretation is consistent with literature suggesting that extra-musical supports, including images, memories, metaphors, and narratives, can play an important role in performers’ interpretive processes and in their subjective experience of nuance, phrasing, and musical character (Héroux, 2016; Héroux et al., 2020; Woody, 2006a,b).

At the same time, the present findings also indicate an important limitation. Although several students ultimately reported that phrase-specific storytelling helped them feel more immersed and more expressive, some also described having difficulty both in generating a narrative image and in realizing that image in sound. This suggests that the challenge of imagery was not simply whether students could imagine a story, but whether they could translate a self-generated, internally meaningful image into concrete performance decisions. In this respect, the present pattern is compatible with prior work by Van Zijl and Luck (2013) and Van Zijl et al. (2014), which suggests that what feels more personal or emotionally convincing to performers does not necessarily coincide with what listeners judge to be more skilled or more effective. Thus, the present results should not be read as showing that imagery was ineffective, but rather that subjective interpretive gain and externally judged sound-level change may emerge on different levels and on different timescales.

Pedagogically, this implies that imagery may be especially valuable for developing ownership, emotional engagement, and interpretive direction, but that it requires explicit support for image-to-sound translation and self-monitoring. As Woody’s framework suggests, effective expressive work depends not only on goal imaging but also on motor production and self-monitoring (Woody, 2003), and McPhail (2025) likewise emphasizes that learners need both a clear sense of the intended outcome and an awareness of the gap between that ideal and their current performance. From this perspective, the pedagogical challenge is not only to encourage vivid imagery, but also to help students specify how a particular character, scene, or narrative trajectory should shape phrasing, dynamics, rubato, articulation, and tone color, and then to evaluate whether those intentions are actually audible in their playing. In short, imagery may be most productive when treated not as an endpoint of subjective immersion, but as an interpretive catalyst for explicitly mapping internal metaphors onto concrete acoustic cues.

### 4.5 Integration of ratings and learner reports

The present findings support the value of using multidimensional expert ratings alongside learner-report measures. In the present study, group differences did not emerge for overall expressiveness or accuracy, but they did emerge for specific performance dimensions and for several aspects of reported practice experience. Had the analysis relied only on a single overall expressiveness score, it would have been more difficult to capture the differentiated pattern observed here—namely, that modeling showed relative strengths in some cue-level outcomes, whereas imagery showed relative strengths in subjective interpretive experience. Such differentiation underscores the importance of examining performance and learner experience across multiple dimensions. More specifically, the results suggest that expert ratings and learner reports may be sensitive to different aspects of expressive development: expert ratings capture whether expressive intentions have been audibly externalized, whereas learner reports provide insight into whether students experienced clearer direction, stronger ownership, or greater engagement during the process of shaping performance. Consequently, these two forms of data should be viewed as complementary sources of evidence, both of which contribute to a more holistic understanding of expressive development.

### 4.6 Pedagogical implications

From a pedagogical perspective, the present findings do not support the conclusion that one approach is universally superior. Rather, they suggest that modeling, structural understanding, and narrative imagery offer different kinds of support and may be most useful when applied strategically in relation to specific pedagogical goals. Modeling appears especially useful when students need a clear external reference for noticing and refining particular expressive cues in sound. Structural understanding may be especially valuable when the goal is to organize performance decisions through harmonic, formal, and phrase-based reasoning. Narrative imagery may be particularly effective for strengthening expressive intention, emotional engagement, and interpretive ownership, even when its immediate sound-level effects are less readily detectable by expert listeners.

Accordingly, these approaches may be most productive when treated as complementary rather than competing tools. One possible sequence is to begin with narrative imagery or structural understanding in order to establish interpretive direction and expressive intention, and then to use modeling selectively to refine how specific cues are realized in sound. Conversely, modeling may also be introduced earlier as a way of sharpening students’ awareness of audible expressive details, after which structural understanding or narrative imagery can help transform those cues into a more personally owned interpretation. In either case, the present findings suggest that modeling is likely to be more productive when it is used selectively (for example, by focusing on one or two musical elements at a time) rather than as an invitation to imitate an entire performance wholesale.

More broadly, the pedagogical issue is not which single approach should replace the others, but how different approaches can be combined so that internally generated interpretive ideas are linked to musically grounded decisions and, ultimately, to audible performance change. In this sense, expressive-practice instruction may be most effective when teachers and students treat these approaches not as fixed methods, but as flexible resources that can be sequenced, combined, and adapted according to the learner’s needs and the specific expressive demands of the musical task.

### 4.7 Limitations and future directions

Several limitations of the present study should be acknowledged. The study used a single short excerpt and a single practice session, so the findings should be understood as short-term and task-specific. All participants were women, which may limit the generalizability of the findings. In addition, although the study was theoretically grounded in goal-image formation, it did not directly measure the quality or clarity of students’ goal images. Future research could therefore examine longer interventions, delayed posttests, transfer tasks, and more direct process measures of intended-performance representation, image-to-sound translation, and self-monitoring during practice.

## Data Availability

The original contributions presented in this study are included in the article/Supplementary material, further inquiries can be directed to the corresponding author.
