# Supplementary material for: Independent practice approaches for expressive piano performance: modeling, structural understanding, and narrative imagery
Source: Front Psychol. 2026 Jun 8;17:1836640. doi: 10.3389/fpsyg.2026.1836640 (PMC13284693; doi:10.3389/fpsyg.2026.1836640)
Supplement: Supplementary file 1 [file Table_1.docx]

Supplementary Table 1

**Supplementary Table 1.** Pretest and posttest means, standard deviations, and change scores (Δ = Post − Pre) by group for expert-rated performance dimensions

| Measure | Group | *n* | Pre | Post | Δ |
| --- | --- | --- | --- | --- | --- |
|  |  |  | *M (SD)* | *M (SD)* | *M (SD)* |
| Phrasing | G1 | 18 | 2.61 (0.56) | 2.87 (0.58) | 0.26 (0.62) |
|  | G2 | 18 | 2.50 (0.73) | 2.89 (0.73) | 0.39 (0.49) |
|  | G3 | 18 | 2.83 (0.66) | 2.78 (0.38) | −0.06 (0.54) |
| Tone color | G1 | 18 | 2.37 (0.70) | 2.70 (0.57) | 0.33 (0.57) |
|  | G2 | 18 | 2.41 (0.58) | 2.50 (0.73) | 0.11 (0.36) |
|  | G3 | 18 | 2.50 (0.57) | 2.22 (0.30) | −0.28 (0.51) |
| Dynamics | G1 | 18 | 2.54 (0.44) | 2.81 (0.63) | 0.28 (0.46) |
|  | G2 | 18 | 2.41 (0.58) | 2.63 (0.71) | 0.22 (0.47) |
|  | G3 | 18 | 2.52 (0.64) | 2.24 (0.36) | −0.28 (0.51) |
| Tempo rubato | G1 | 18 | 2.65 (0.67) | 3.11 (0.47) | 0.46 (0.49) |
|  | G2 | 18 | 2.43 (0.76) | 2.83 (0.71) | 0.41 (0.53) |
|  | G3 | 18 | 2.46 (0.76) | 2.44 (0.43) | −0.02 (0.64) |
| Balance | G1 | 18 | 2.70 (0.53) | 2.74 (0.42) | 0.04 (0.39) |
|  | G2 | 18 | 2.56 (0.62) | 2.70 (0.55) | 0.15 (0.54) |
|  | G3 | 18 | 2.41 (0.54) | 2.56 (0.36) | 0.15 (0.53) |
| Pedaling | G1 | 18 | 2.28 (0.61) | 2.20 (0.47) | −0.07 (0.58) |
|  | G2 | 18 | 2.70 (0.53) | 2.81 (0.65) | 0.11 (0.26) |
|  | G3 | 18 | 2.41 (0.44) | 2.46 (0.33) | 0.06 (0.49) |
| Articulation | G1 | 18 | 2.54 (0.47) | 2.87 (0.63) | 0.33 (0.54) |
|  | G2 | 18 | 2.56 (0.50) | 2.74 (0.44) | 0.19 (0.26) |
|  | G3 | 18 | 2.28 (0.43) | 2.20 (0.26) | −0.07 (0.37) |
| Overall expressiveness | G1 | 18 | 2.50 (0.83) | 2.54 (0.46) | 0.04 (0.81) |
|  | G2 | 18 | 2.28 (0.71) | 2.67 (0.69) | 0.39 (0.38) |
|  | G3 | 18 | 2.65 (0.84) | 2.61 (0.42) | −0.04 (0.74) |
| Accuracy | G1 | 18 | 2.48 (0.50) | 2.61 (0.40) | 0.13 (0.53) |
|  | G2 | 18 | 3.20 (0.62) | 2.96 (0.57) | −0.24 (0.36) |
|  | G3 | 18 | 2.80 (0.62) | 2.69 (0.33) | −0.11 (0.62) |

*Note*. G1 = modeling, G2 = structural, G3 = imagery.
