# Supplementary material for: Independent practice approaches for expressive piano performance: modeling, structural understanding, and narrative imagery
Source: Front Psychol. 2026 Jun 8;17:1836640. doi: 10.3389/fpsyg.2026.1836640 (PMC13284693; doi:10.3389/fpsyg.2026.1836640)
Supplement: Supplementary file 2 [file Table_2.docx]

Supplementary Table 2

**Supplementary Table 2.** Time and Time × Group effects from pretest–posttest mixed-design ANOVAs for expert-rated performance dimensions

| Measure | Effect | *F* | *df* | *p* | η_p_² |
| --- | --- | --- | --- | --- | --- |
| Phrasing | Time | 6.903 | 1, 51 | .011 | .119 |
|  | Time × Group | 3.080 | 2, 51 | .055 | .108 |
| Tone color | Time | 0.692 | 1, 51 | .409 | .013 |
|  | Time × Group | 7.154 | 2, 51 | .002 | .219 |
| Dynamics | Time | 1.271 | 1, 51 | .265 | .024 |
|  | Time × Group | 7.229 | 2, 51 | .002 | .221 |
| Tempo rubato | Time | 14.041 | 1, 51 | <.001 | .216 |
|  | Time × Group | 4.028 | 2, 51 | .024 | .136 |
| Balance | Time | 2.765 | 1, 51 | .102 | .051 |
|  | Time × Group | 0.307 | 2, 51 | .737 | .012 |
| Articulation | Time | 7.200 | 1, 51 | .010 | .124 |
|  | Time × Group | 4.650 | 2, 51 | .014 | .154 |
| Overall expressiveness | Time | 2.021 | 1, 51 | .161 | .038 |
|  | Time × Group | 2.076 | 2, 51 | .136 | .075 |
| Accuracy | Time | 1.135 | 1, 51 | .292 | .022 |
|  | Time × Group | 2.434 | 2, 51 | .098 | .087 |

*Note*. η_p_² = partial eta squared. Pedaling was excluded from inferential analyses because interrater reliability was low.
