# Supplementary material for: Independent practice approaches for expressive piano performance: modeling, structural understanding, and narrative imagery
Source: Front Psychol. 2026 Jun 8;17:1836640. doi: 10.3389/fpsyg.2026.1836640 (PMC13284693; doi:10.3389/fpsyg.2026.1836640)
Supplement: Supplementary file 3 [file Table_3.docx]

Supplementary Table 3

**Supplementary Table 3.** Tukey HSD post hoc comparisons of change scores for expert-rated performance dimensions showing significant Time × Group interactions

| Measure | Pairwise comparison | Mean difference | *SE* | *p* | 95% CI |
| --- | --- | --- | --- | --- | --- |
| Tone color | G1 − G3 | 0.611 | 0.164 | .001 | [0.216, 1.006] |
|  | G2 − G3 | 0.389 | 0.164 | .054 | [−0.006, 0.784] |
|  | G1 − G2 | 0.222 | 0.164 | .370 | [−0.173, 0.617] |
| Dynamics | G1 − G3 | 0.556 | 0.161 | .003 | [0.167, 0.944] |
|  | G2 − G3 | 0.500 | 0.161 | .009 | [0.112, 0.889] |
|  | G1 − G2 | 0.056 | 0.161 | .936 | [−0.333, 0.444] |
| Tempo rubato | G1 − G3 | 0.481 | 0.186 | .033 | [0.033, 0.930] |
|  | G2 − G3 | 0.426 | 0.186 | .066 | [−0.022, 0.874] |
|  | G1 − G2 | 0.056 | 0.186 | .952 | [−0.393, 0.504] |
| Articulation | G1 − G3 | 0.407 | 0.135 | .011 | [0.081, 0.734] |
|  | G2 − G3 | 0.259 | 0.135 | .144 | [−0.067, 0.586] |
|  | G1 − G2 | 0.148 | 0.135 | .521 | [−0.178, 0.475] |

*Note*. Comparisons are based on change scores (Δ = Post − Pre). Mean differences are calculated as the first group minus the second group. G1 = modeling, G2 = structural, G3 = imagery. CI = confidence interval.
